# Responses of biomass accumulation and nutrient utilization along a phosphorus supply gradient in *Leymus chinensis*

**DOI:** 10.1038/s41598-023-31402-4

**Published:** 2023-04-06

**Authors:** Huijun Li, Yutong Hu, Gongshe Liu, Jiandong Sheng, Wentai Zhang, Hongmei Zhao, Hongliang Kang, Xiaoguo Zhou

**Affiliations:** 1 College of Resources and Environment, Xinjiang Agricultural University, Urumqi, 830052 Xinjiang China; 2 Xinjiang Key Laboratory of Soil and Plant Ecological Processes, Urumqi, 830052 Xinjiang China; 3 The Research Center of Soil and Water Conservation and Ecological Environment, Chinese Academy of Sciences and Ministry of Education, Yangling, 712100 Shanxi China; 4 University of Chinese Academy of Sciences, Beijing, 100049 China; 5 Key Laboratory of Plant Resources, Institute of Botany, Chinese Academy of Sciences, Beijing, 100093 China; 6 State Key Laboratory of Erosion and Dryland Agriculture On the Loess Plateaus, Institute of Soil and Water Conservation, Northwest A&F University, Yangling, 712100 China

**Keywords:** Ecology, Grassland ecology

## Abstract

Phosphorus (P) deficiencies are widespread in calcareous soils. The poor availability of nitrogen (N) and P in soils often restricts crop growth. However, the effects of P addition on plant growth and plant nutrient transport changes during the establishment of *Leymus chinensis* fields in Xinjiang are not clear. We investigated the responses of *Leymus chinensis* biomass and nutrient absorption and utilization to changes in soil N and P by adding P (0, 15.3, 30.6, and 45.9 kg P ha^−1^ year^−1^) with basally applied N fertilizer (150 kg N ha^−1^ year^−1^). The results showed that (a) Principal component analysis (PCA) of biomass, nutrient accumulation, soil available P, and soil available N during the different periods of *Leymus chinensis* growth showed that their cumulative contributions during the jointing and harvest periods reached 95.4% and 88%, respectively. (b) Phosphorus use efficiency (PUE) increased with the increase of P fertilizer gradient and then decreased and the maximum PUE was 13.14% under moderate P addition. The accumulation of biomass and nutrients in *Leymus chinensis* can be effectively improved by the addition of P fertilizer at 30.6 kg ha^−1^. Different P additions either moderately promoted or excessively inhibited *Leymus chinensis* growth and nutrient utilization.

## Introduction

**Figure 1:**
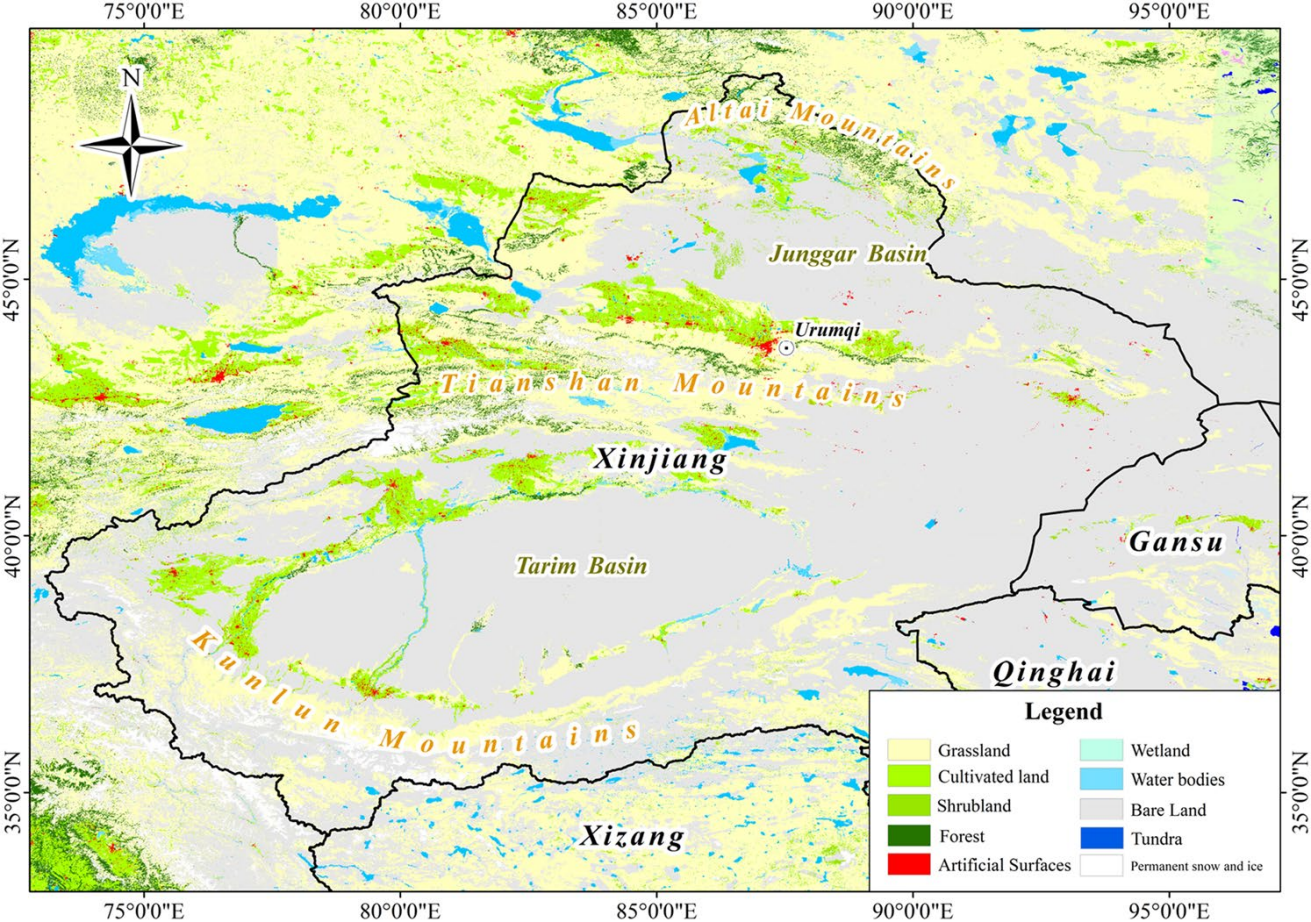
Types of land cover in Xinjiang of China. The land cover classification is based on the GlobeLand30 2020 (http://www.globallandcover.com/). The graph is drawn by ArcMap 10.7.

China's natural grassland area is approximately 4 million km^2^, accounting for 41.7% of China's total land area^1,2^. Grassland ecosystems, asn important type of natural ecosystem, have high geographical value because they maintain ecological balance, influence the regional economy and affect human history^3,4^. In addition, they also play important roles in windbreak provision, sand fixation, soil improvement, climate regulation and inter-regional biodiversity^3^. Xinjiang is one of the five pastoral areas in China, and its grassland area is third only to that in Inner Mongolia and Tibet.^5^ (Fig. 1) (obtained from GlobeLand30 2020; http://www.globallandcover.com/). However, in recent years, with the rapid development of animal husbandry and the intrusion of human activities into natural spaces, grassland areas have decreased dramatically, their productivity have been reduced^6^. To quickly restore degraded grasslands, numerous researchers have carried out active and effective restoration strategies. For example, Zhang et al.^7,8^ found that *Leymus chinensis* (Trin.) Tzvel has more advantages than other grass forages when used for artificial grassland restoration and degraded grassland improvement. *Leymus chinensis* is a perennial rhizomatous grass that is widely distributed in the eastern part of the Eurasian steppe belt, in the eastern part of the Songnen Plain, and on the Inner Mongolia Plateau in China^9^. Many studies have shown that *Leymus chinensis* has good palatability, a strong regeneration capacity, long green-holding periods, and large leaf volumes^10^. Because *Leymus chinensis* is rich in the crude fats, crude proteins, and crude fibres required for the growth of cattle and sheep, it is known as the fine grain of forage^11^. Because of its strong adaptability to different living environments and its salinity, infertility, trampling, cold, and drought resistance, *Leymus chinensis* is a high-quality forage grass, especially for grassland restoration^7,12^, and it has great significance for the management and improvement of grassland ecosystems in China^13^.

During agricultural production, fertilizers are consistently used to improve crop yields, but the overuse of chemical fertilizers has led to the introduction of excessive quantities of nutrients and to eutrophication in many countries and regions of the world, which eventually lead to the depletion of nutrients. In particular, phosphorus (P) resources, such as inorganic phosphate (Pi), can be absorbed and used by crops^14,15^. Phosphate is involved in energy transfer and the synthesis of macromolecules in cells. A large number of elements and nutrient resources are required for the growth and development of plants^16–18^. Phosphate not only is a component of many important organic compounds in plants but also participates in different forms in the various metabolic processes active in plants^19–22^, so it is necessary to supplement soils with Pi in appropriate amounts during plant growth^23,24^. However, during agricultural production and the growth of plants in the natural environment, only 10–20% of the available Pi is utilized during the growing season, and the vast majority of Pi is easily fixed by adsorption to soil particles due to the low mobility of P^25^. In calcareous soils controlled by calcium and magnesium, phosphate ions in the soil solution are in the form of HPO4^2−^, which chemically interacts with the exchangeable Ca^+^ on soil colloids to produce Ca-P compounds^26,27^. Due to widespread soil Pi deficiencies in the grasslands of northern China, Pi availability is closely related to the growth and development of livestock and forage resources^28,29^. A study by Xu et al.^30^ on southern grasslands showed that the continued application of calcium-magnesium and P fertilizer to grazed grasslands over two years had a significant effect on forage yield and quality in the region. He et al.^31^ showed that during the early period of growth, forage grasses took up more Pi than was available in the surface soils, and the mass fraction of soil microbial Pi decreased with an increasing soil available Pi content. Roberts et al.^22^ showed that in grassland ecosystems, the growth and development of most species are often limited by nutrients, especially P, which tends to be the least active major nutrient in most soils^32^.

To date, there have been few studies on *Leymus chinensis* in Xinjiang and few reports on the restoration and improvement of artificial *Leymus chinensis* lands by Pi amendment in Xinjiang. There is an urgent need to develop a greater number of forage and livestock areas in the Xinjiang region due to the high demand for forage grasses and the destruction of the ecological balance of grasses due to overgrazing. Therefore, in this study, the predominantly calcareous soils of the Xinjiang grasslands were selected for studying *Leymus chinensis* growth. The growth characteristics and nutrient utilization of *Leymus chinensis* in response to different P application conditions were quantitatively analysed by pot experiments, and the optimal P application for improving the biomass and phosphorus utilization efficiency (PUE) of *Leymus chinensis* was determined to provide a theoretical basis for future plantings of artificial *Leymus chinensis* grasslands and for the restoration of degraded grasslands. We hypothesize that (1) Increased application of phosphorus fertilizer under low phosphorus conditions can promote the absorption of phosphorus in *Leymus chinensis*, thus breaking the nutrient restriction of phosphorus in *Leymus chinensis*. (2) Within a certain range of phosphorus application, the PUE may increase with the amount of phosphorus fertilizer added, but will not continue to increase when it reaches a certain threshold.

## Results

### Responses of biomass accumulation and nutrient allocation to different P supply geadient

#### Effect of P supply gradient on biomass

**Figure 2:**
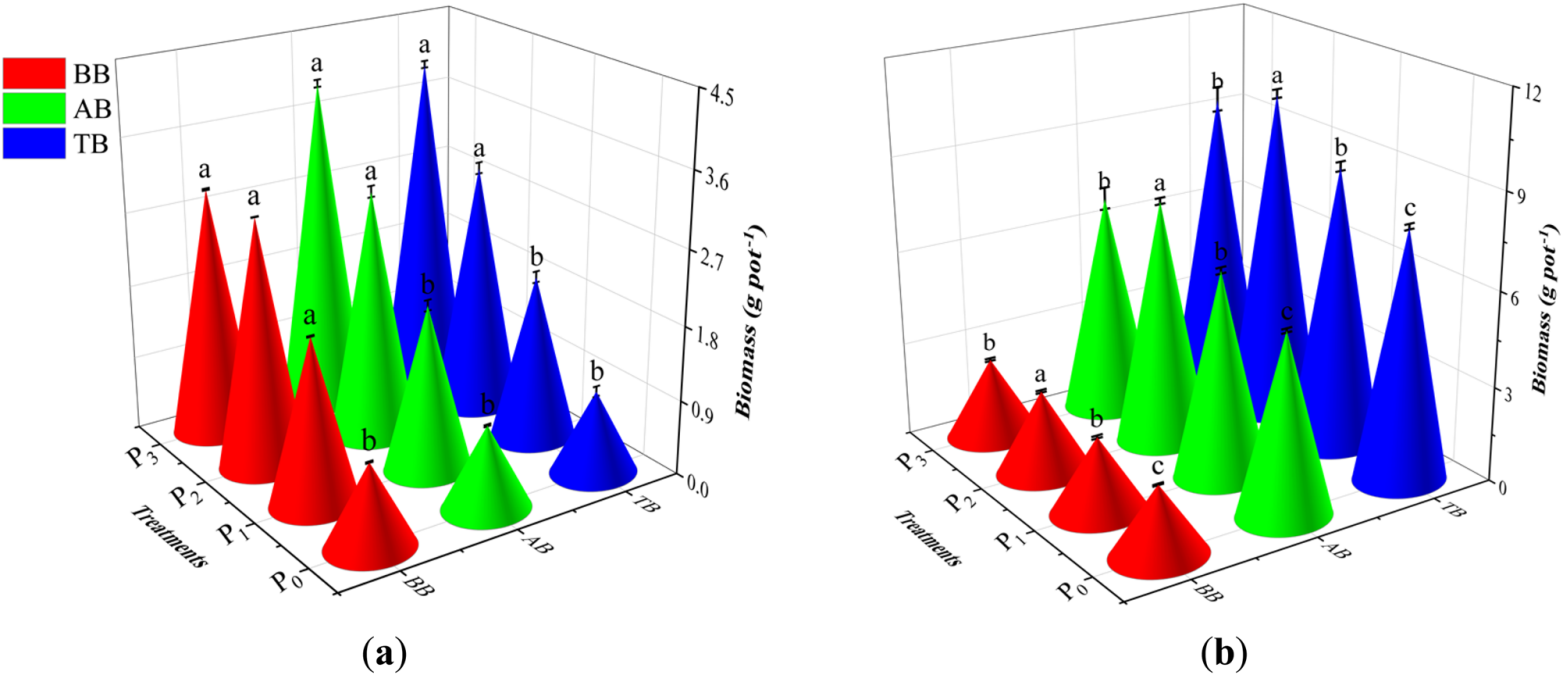
Effects of P supply gradient on biomass accumulation during the (**a**) jointing period and (**b**) harvesting period. TB, total biomass; AB, aboveground biomass; BB, belowground biomass; P_0_, no P; P_1_, 15.3 kg ha^−1^; P_2_, 30.6 kg ha^−1^; P_3_, 45.9 kg ha^−1^. Lowercase letters indicate significant differences among treatments at *p* < 0.05 (one-way ANOVA with LSD test, n = 3). The error bars indicate standard error of the mean.

The aboveground biomass, belowground biomass, and total biomass of *Leymus chinensis* showed an increasing and then decreasing trend with increasing P applications during the jointing period and the harvest period and the aboveground biomass, belowground biomass, and total biomass reached a maximum at a 30.6 kg ha^−1^ P application (*p* < 0.05; Fig. 2). The biomass of each component was lowest at P_0_, and there was a significant difference between the P_0_ and P_2_ treatments. The accumulation of aboveground biomass was greater than that of belowground biomass during the jointing period and harvest period, and in terms of the effect on biomass accumulation, the P application treatments were ordered P_2_ > P_3_ > P_1_ > P_0_.

During the jointing period, the aboveground biomass differed significantly between the P_2_, P_3_, P_1_, and P_0_ treatments. It increased by 53.75%, 163.75%, and 155% along the P application gradient (P_1_, P_2_ and P_3_) compared with P_0_. The accumulation of belowground biomass was significantly different in the P_1_, P_2_, and P_3_ treatments compared with the P_0_ treatment, increasing by 12.44%, 22.28%, and 22.28%, respectively. The trend of total biomass accumulation was consistent with that of the accumulation of aboveground biomass, which was significantly different between the P_2_ and P_0_ treatments and 136.36% higher in the P_2_ treatment than in P_0_. During the harvest period, the accumulation of aboveground biomass was significantly different between P_0_ and each P application, and the accumulation of aboveground biomass increased by 13.80%, 35.29%, and 21.84% along the gradient. P_2_ was significantly different compared with P_0_, P_1_, and P_3_, and the accumulation of belowground biomass was significantly different between each P application along the gradient and P_0_. The belowground biomass increased by 8.23%, 19.97%, and 15.55% in P_0_, P_2_, and P_3_ compared with P_1_; the accumulation of total biomass was significantly different in P_0_ compared with P_1_, P_2_, and P_3_; and the accumulation of total biomass increased by 12.26%, 31.05% and 20.10% at each P application along the gradient compared with P_0_.

**Figure 3:**
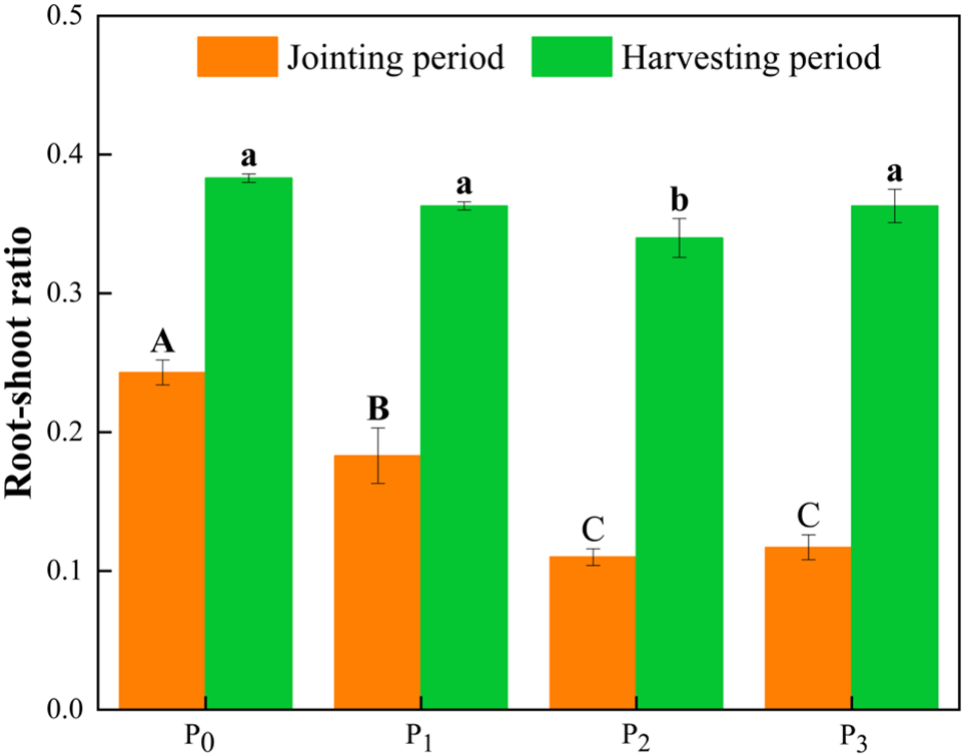
Effects of P supply gradient on biomass allocation. Different uppercase letters indicate significant differences in root-shoot ratio of *Leymus chinensis* at jointing period (*p* < 0.05, one-­way ANOVA, n = 3), while lowercase letters indicate significant differences in root-shoot ratio of *Leymus chinensis* at harvesting period (*p* < 0.05, one-­way ANOVA, n = 3).

As shown in Fig. 3, the accumulation of aboveground biomass reached 4.64–9.77% of the overall aboveground biomass accumulation over the reproductive period, while the remaining 95.36–90.23% of the aboveground biomass accumulation occurred mainly after the jointing period. The accumulation of aboveground biomass occurred mainly during the early period of *Leymus chinensis* growth when it reached 90% at 30.6–45.9 kg ha^−1^ P application, and the accumulation of belowground biomass gradually increased with the progression of the reproductive period. The biomass distribution for each group was not greatly affected by the P applications. The root-shoot ratio of *Leymus chinensis* during the harvest period was the lowest at a P application of 30.6 kg ha^−1^, which was significantly different from that in P_0_, P_1_, and P_3_ (*p* < 0.05), and the root-shoot ratio showed a decreasing and then increasing trend with the increase in P application along the gradient. Compared with that in P_0_, the root-shoot ratio of *Leymus chinensis* under P application (P_1_, P_2_, and P_3_) decreased by 5.22%, 10.53%, and 5.26% (*p* < 0.05; Fig. 3), respectively.

#### Effect of P supply gradient on nutrient accumulation in *Leymus chinensis*

**Figure 4:**
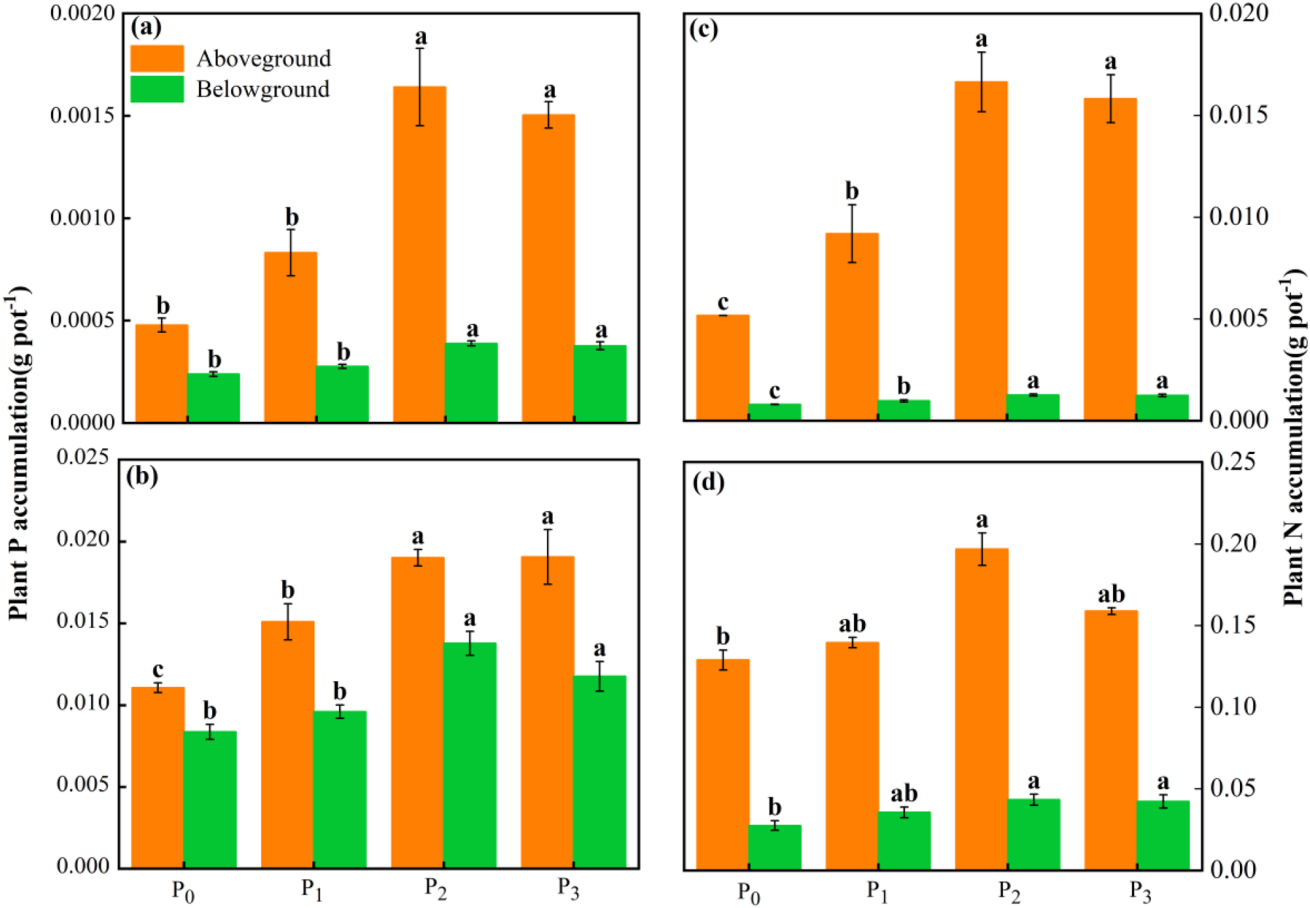
Nutrient accumulation aboveground and belowground under P supply gradient. Plant P accumulation during the (**a**) jointing period and (**b**) harvest period. Plant N accumulation during the (**c**) jointing period and (**d**) harvest period. The different lowercase letters mean the significant difference among treatments at *p* < 0.05 (one­way ANOVA with LSD test). The error bars indicate standard error of the mean with the number of observation (n = 3).

P accumulation in the belowground and aboveground parts of *Leymus chinensis* showed a trend of increasing and then decreasing with increasing P application, and the P accumulation in the belowground and aboveground parts of *Leymus chinensis* significantly increased with the application of 30.6 ~ 45.9 kg ha^−1^ P (*p* < 0.05; Fig. 4a,b). The accumulation of P was greatest for the P_2_ and P_3_ treatment during the jointing period. Moreover, the accumulation of P in all parts of *Leymus chinensis* significantly increased for P_2_ and P_3_ compared with P_0_ and P_1_. Specifically, the accumulation of P in the aboveground parts increased by 97.33% and 80.94% in P_2_ and P_3_, respectively, compared with P_1_, and the accumulation of P belowground significantly increased for P_0_ compared with the P_2_ and P_3_ treatments. The accumulation of aboveground P increased significantly in P_2_ and P_3_ compared with P_0_, with the largest increase occurring in P_2_. The accumulation of aboveground P increased with increasing P application during the harvest period, reaching a maximum in P_3_. In addition, the accumulation of aboveground P increased significantly in each P application along the gradient compared with P_0_, increasing by 36.44%, 71.83%, and 72.29%, respectively.

The aboveground, belowground, and total N accumulation of *Leymus chinensis* showed a trend of increasing and then decreasing with the increase in P application during the jointing period, showing the highest values in the P_2_ treatment (*P* < 0.05; Fig. 4c), and the aboveground, belowground and total N accumulation increased significantly for each P application treatment compared with P_0_. The belowground accumulation of N increased by 21.98%, 57.81%, and 55.17% for each P application treatment along the gradient compared with P_0_. The accumulations of total N in P_2_ and P_3_ were significantly higher than that in the low P treatment (P_1_) by 76.09% and 67.81%, respectively. The accumulation of aboveground N, belowground N, and total N was not significantly different among the P application treatments during the harvest period. The accumulation of N in each part showed a trend of increasing and then decreasing with increasing P application, and the accumulation of N was highest in P_3_ (*p* < 0.05; Fig. 4d). The accumulation of total N increased significantly in P_2_ and P_3_, and it increased by 11.98%, 53.53%, and 28.59% in the P application treatments along the gradient compared with P_0_.

#### The relationship between the observed indices

**Figure 5:**
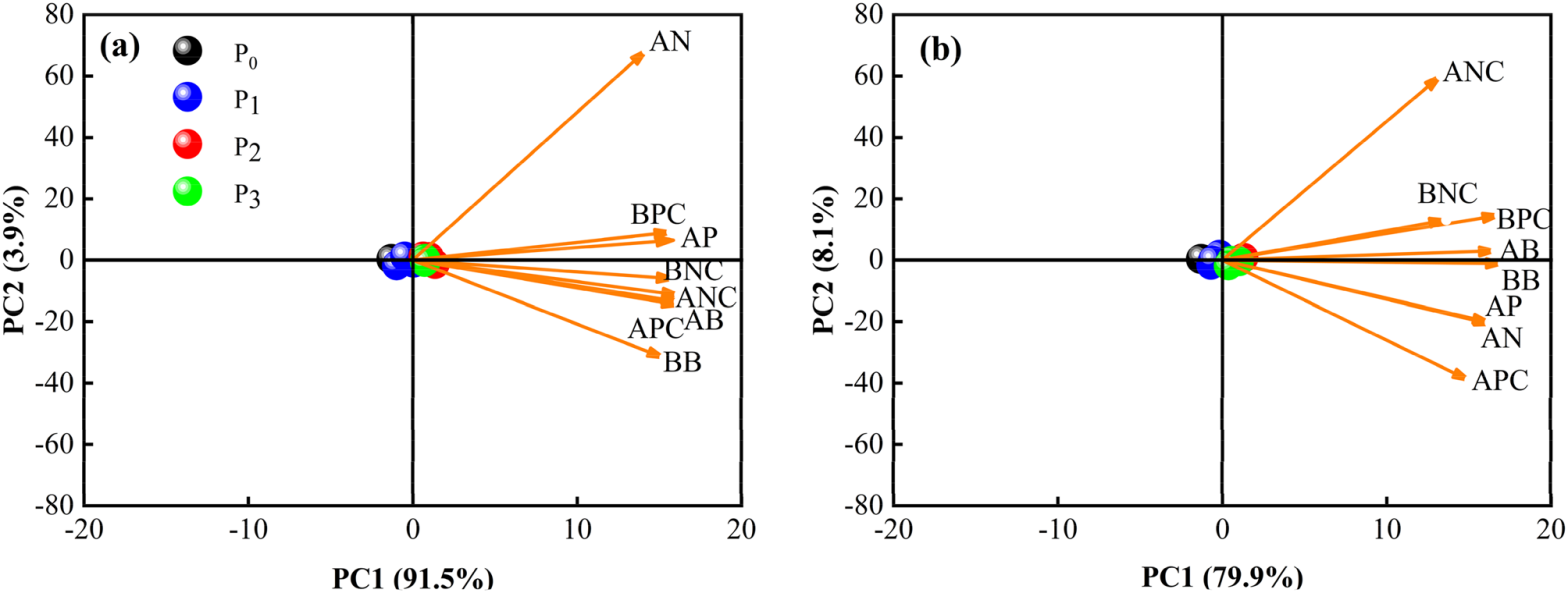
PCA of nutrient accumulation and biomass under different P supply gradient during the (**a**) jointing period and (**b**) harvest period. Notes: AN: available nitrogen; AP: available phosphorus; AB: Aboveground biomass; BB: Belowground biomass; BPC: Belowground phosphorus content; APC: Aboveground phosphorus content; BNC: Belowground nitrogen content; ANC: Aboveground nitrogen content.

The two principal components that could explain the effect of P application on the biomass, nutrients, soil available P, and available N accumulation of *Leymus chinensis* were extracted and plotted using the criterion of an eigenvalue > 1. These results indicate that P application in the presence of sufficient N fertilization has a positive effect on the biomass, nutrient accumulation, soil available P, and available N accumulation of *Leymus chinensis*. The amount of variation explained by PC1 was 91.5% and that by PC2 was 3.9% for the jointing period, with a cumulative contribution of 95.4%. Therefore, the first two principal components reflected the true influence of P applications on the indicators of *Leymus chinensis* biomass and nutrient accumulation and soil available P and available N (*p* < 0.05; Fig. 5a). For the harvest period, PC1 and PC2 explained 88.0% of the correlation between P application and *Leymus chinensis* biomass accumulation, *Leymus chinensis* N and P accumulation, and soil available P and available N accumulation (*p* < 0.05; Fig. 5b).

#### Dependence of nutrient uptake capacity on soil nutrient concentrations

**Figure 6:**
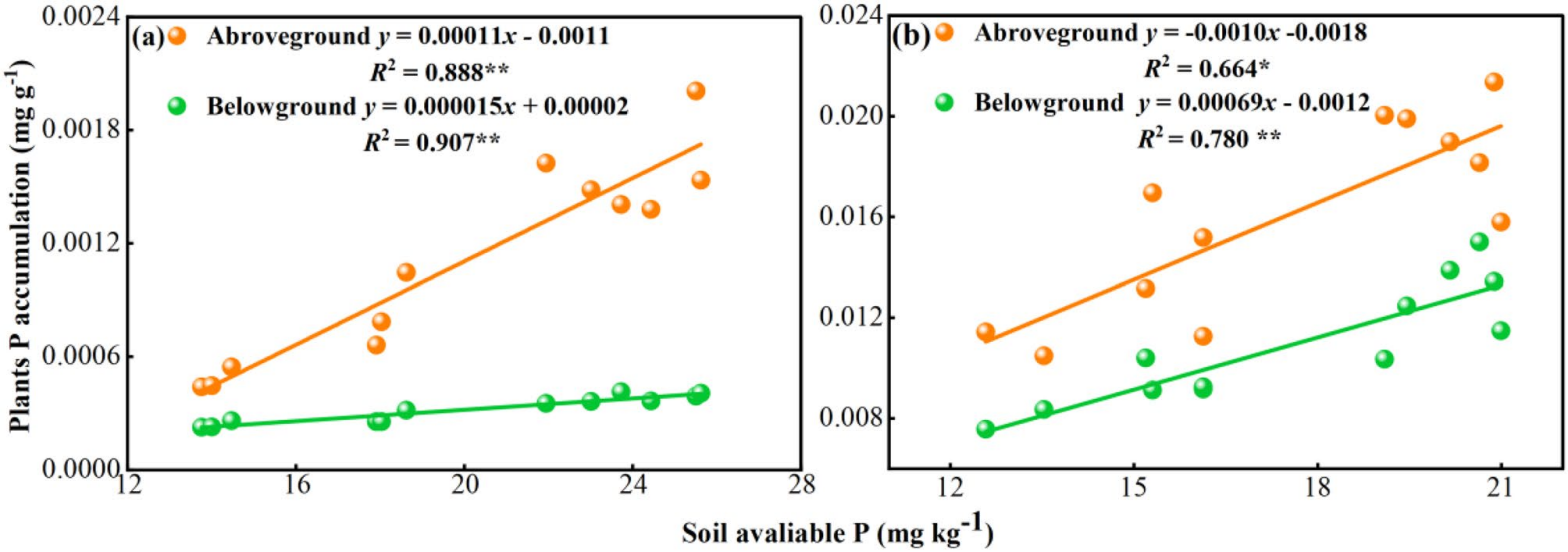
Correlation of plant P accumulation and soil available P during the (**a**) jointing period and (**b**) harvest period. *R*^2^ is given. ** indicate the level of significance for the single-exponential regression: *p < 0.05; **p < 0.01 (n = 3).

The P accumulation in plants was positively correlated with the accumulation of available P in the soil during each growth period of *Leymus chinensis* (*p* < 0.05; Fig. 6), indicating that the changes in available P accumulation in the soil were closely related to the P uptake in each tissue of *Leymus chinensis*. The relationship between P uptake by the aboveground and belowground parts and the amount of available P in the soil was stronger during the jointing period than during the harvest period, and the fit values of *R*^2^ = 0.888 and *R*^2^ = 0.907 were higher than those of *R*^2^ = 0.815 and 0.884 during the harvest period.

**Figure 7:**
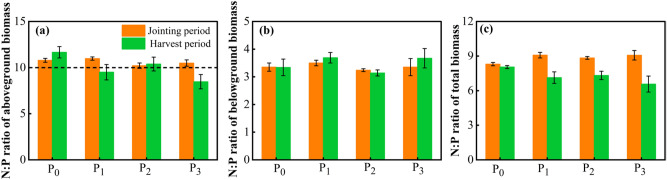
Relationship between P supply gradient and N:P ratios in (**a**) aboveground biomass, (**b**) belowground biomass, and (**c**) total biomass. The error bars indicate standard error of the mean with the number of observation (n = 3).

As shown in Fig. 7, the differences in N:P ratios between the aboveground and belowground parts and the total plant N:P ratios between treatments were not significant, and the total N:P ratio of the belowground parts and plants of *Leymus chinensis* was less than 10. The N:P ratio of the aboveground parts fluctuated around 10, with values > 10 for P_0_ and P_2_ (Fig. 7a). In contrast, in the belowground parts of *Leymus chinensis*, the N:P ratio was the lowest, with values less than 5 (Fig. 7b). The N:P ratio of *Leymus chinensis* was close to 10 (Fig. 7c), and the N:P ratio decreased between growth stages in the order of jointing period > harvest period. With an increase in P application, the N:P ratio showed a decreasing trend for *Leymus chinensis* during the harvest period, decreasing by 11.46%, 9.15%, and 18.38% in P_1_, P_2_, and P_3_, respectively, compared with P_0_.

**Figure 8:**
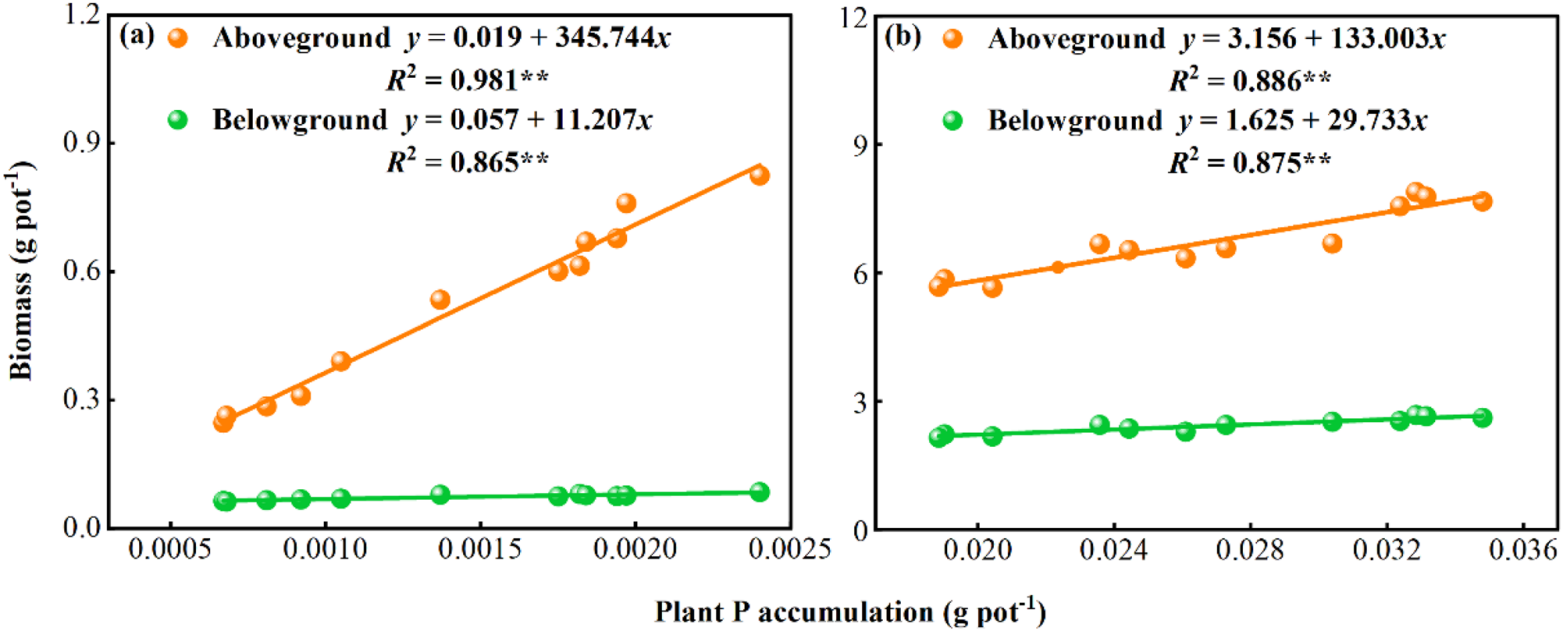
Fitted relationships between P accumulation and biomass in plants. The relationships for plant biomass during the (**a**) jointing period and (**b**) harvesting period. ** indicate the level of significance for the single-exponential regression: **p < 0.01 (n = 3).

A highly significant linear regression relationship between biomass and plant P accumulation was fitted for *Leymus chinensis* (*p* < 0.05; Fig. 8a,b). The fitted equations for the aboveground biomass versus plant P accumulation and the belowground biomass versus plant P accumulation during the jointing period for *Leymus chinensis* were *y* = 0.019 + 345.744*x* (*R*^2^ = 0.981) and y = 0.057 + 11.207*x* (*R*^2^ = 0.865), respectively, and those during the harvest period were *y* = 3.156 + 133.003*x* (*R*^2^ = 0.886) and *y* = 1.625 + 29.733*x* (*R*^2^ = 0.875), respectively. Overall, the *R*^2^ values for *Leymus chinensis* P accumulation and aboveground biomass were higher than those for the relationships between the accumulation of belowground biomass and *Leymus chinensis* P accumulation.

#### Effect of P supply gradient on PFPP and PUE

The phosphate fertilizer partial productivity (PFPP) varied considerably during the different periods of *Leymus chinensis* growth, and it showed a decreasing trend with increasing P application during the same growth period, reaching a maximum in the 15.3 kg ha^−1^ P treatment, with values of 9.44 g g^−1^ and 173.83 g g^−1^ for the jointing period and harvest period, respectively (Table 1). During the jointing period, the PFPP decreased by 18.18% and 47.42% in P_2_ and P_3_, respectively, compared with P_1_, while during the harvest period, the PFPP decreased significantly among the treatments and was lowest in P_3_, with decreases of 41.01% and 64.24% for P_2_ and P_3_, respectively, compared with P_1_. The PUE increased and then decreased along the P application gradient during the jointing period and the harvest period of *Leymus chinensis*, the PUE for each growth period reached a maximum value at P_2_, and the lowest PUE for each growth period of *Leymus chinensis* occurred at P_3_ (*p* < 0.05; Table 1). During the jointing period, the difference in PUE along the P application gradient was not significant, and the PUE in P_2_ was comparable to that in P_1_. The PUE increased by 68.51% between P_2_ and P_1_ and decreased by 0.46% between P_3_ and P_0_. In contrast, in the harvest period, the PUE was significantly different between P_2_ and P_3_.

**Table 1:** Nutrient accumulation in the aboveground and belowground parts of *Leymus chinensis* under P supply gradient. PFPP, phosphate fertilizer partial productivity; PUE, phosphorus utilization efficiency. Lowercase letters in the same column indicate significant differences between treatments (*p* < 0.05, one­way ANOVA, n = 3). ‘−’ mean not measured.

Treatment	Jointing period		Harvesting period	
	PFPP(g g^−1^)	PUE (%)	PFPP(g g^−1^)	PUE (%)
P_0_	–	–	–	–
P_1_	9.44a	0.77a	173.83a	10.30ab
P_2_	7.73ab	1.29a	102.53b	13.14a
P_3_	4.97b	0.76a	62.17c	7.46b

## Discussion

### Effect of phosphorus supply gradient on the growth of *Leymus chinensis*

The results of this study showed that the biomass accumulation of *Leymus chinensis* increased significantly under each P application treatment, and the best accumulation of biomass in each fraction was achieved under 30.6 kg ha^−1^ P application. There was a close relationship between crop biomass and the nutrient content of the soil (Fig. 5). The nutrients required for crop growth come from the nutrients in the soil, but when the nutrients in the soil are not sufficient to meet the crop growth requirements, they need to be added artificially^33^. For example, crops adapt to nutrient deficiencies by changing the amount of nutrients allocated to each organ, as shown by changes in biomass accumulation and root-to-crown ratios^34^.

The results of this study showed that additional P fertilization significantly increased the accumulation of belowground biomass in *Leymus chinensis*, indicating that additional P fertilization improves crop root growth (Fig. 2a,b), which may be due to changes caused by the stimulatory effect of P addition on the plant growth process. P affects crops by participating in the synthesis of macromolecules and by contributing to a variety of important metabolic processes, which in turn affects crop yield^35^. However, the distribution of biomass among the various parts of the crops was not affected by the amount of P applied. Zhang et al.^36^ showed that the proportion of accumulated biomass in each part of maize does not change with the amount of P applied, and the proportions of accumulated biomass are generally similar among growth periods, while changes in the amount of P applied to affect only the amount of accumulated biomass. This indicates that, within a reasonable range of P applications, supplementing the P in the soil through artificial P amendments can promote the growth and development of the crop. Combined with the observed effect of P applications on the nutrient uptake and P fertilizer utilization in *Leymus chinensis*, these results further clarified that nutrient uptake was better at a P application rate of 30.6 kg ha^−1^, which in turn promoted the growth of *Leymus chinensis*.

### Effect of different phosphorus supply gradient on the nutrient uptake and N:P ratios of *Leymus chinensis*

During the growth of *Leymus chinensis*, nutrient uptake by each organ shows a dynamic process, and with the progression of the reproductive period, each organ has different nutrient requirements at different growth periods^29,37^. The amount of N and P accumulation in each organ of the plant can characterize the ability of the plant to absorb and utilize nutrients in the soil. This study showed that with an increase in P application, both P and N accumulation in *Leymus chinensis* increased and then decreased, and the nutrient accumulation levels reached a maximum value at a P application rate of 30.6 kg ha^−1^, although there was also an increase under a P application rate of 45.9 kg ha^−1^. However, the difference was not significant compared with the 30.6 kg ha^−1^ P treatment, and nutrient accumulation even tended to decrease slightly under high-P conditions, indicating that the addition of P to the soil under low-initial P conditions helped promote biomass accumulation, while under high-P conditions, the growth and development of plants were negatively affected. Studies have shown that the plant N:P ratio can be an important indicator for evaluating crop limitations of N and P accumulation; crop growth was clearly N limited when N:P < 10 and P limited when N:P > 20^38^. In the range of P_0_ ~ P_2_, the accumulation of P in the shoot of *Leymus chinensis* increased with the increase of P application, which indicated that the application of P fertilizer promoted the utilization capacity of P fertilizer and intensified the demand for P in *Leymus chinensis*. The P limitation of *Leymus chinensis* gradually changed into N limitation with the increase of P application. The results of this study showed that in the aboveground parts of *Leymus chinensis*, the N:P ratio was > 10 among all the treatments except for the P_1_ and P_3_ treatments at harvest time, indicating that *Leymus chinensis* was less affected by N and P accumulation under P application conditions. In contrast, the N:P ratio < 10 in the root system of *Leymus chinensis* indicated that root growth was limited by N and P accumulation, but overall, the N:P ratio was close to 10, indicating that *Leymus chinensis* was not affected by N and P accumulation. It has also been shown that the average N:P ratio of terrestrial plants under natural conditions is 12–3^39^. In contrast, the N:P ratio obtained in this study was 8 ~ 12, which may differ greatly due to the geographical distribution of the terrestrial plants in this study because most of the soils in Xinjiang are calcareous and have low fertility, thus decreasing the N:P of local terrestrial plants. When soil N:P accumulation inhibits the growth of crops, proper manipulation of the soil N:P ratio will further promote the growth and development of crops^40^.

### Effect of different phosphorus supply gradient on P fertilizer utilization

The results of this study showed that the accumulation of aboveground nutrients was higher than that of belowground nutrients throughout the reproductive period of *Leymus chinensis*, which is similar to the results of Marque et al.^41^. The accumulation of nutrients in tobacco plants was promoted by P application, and the accumulation of nutrients in the leaves of tobacco plants was higher than that in the roots. During each reproductive period of *Leymus chinensis*, with an increase in P application, the aboveground, belowground and total P accumulation showed an increasing and then decreasing trend, indicating the promotion of nutrient uptake and accumulation in the range of 0–30.6 kg ha^−1^ P application, although the accumulation of P also increased under P_3_ compared with P_0_. However, considering economic efficiency and environmental issues, the optimal P application should be approximately 30.6 kg ha^−1^ P in future experiments. This is similar to the results of Xie et al.^42^, who showed that the accumulation and uptake of P from soils can be promoted by P treatment. Other studies have shown that the accumulation of P in wheat plants increases with an increase in P application during all fertility periods within a certain range of P applications^43^. The results of this study showed that the uptake of elements such as N, P, and K involves dynamic processes that change during the fertile period, and the highest accumulation of each nutrient and the maximum amount of P accumulation were reached during the harvest period in *Leymus chinensis*. This is contrary to the results of the investigation by Peng et al.^29^ on the dynamic change patterns of P in *Leymus chinensis* pastures. Their research showed that the P accumulation of *Leymus chinensis* was highest during the spring growing period because of the preferential transport of P to the growth organs of the plants. In the present study, the P accumulation in the aboveground and belowground parts of *Leymus chinensis* increased with the progression of the reproductive period because of P addition, and P accumulation decreased during the harvest period.

The results of this study indicated that the PFPP decreased gradually with increasing P applications (15.3 ~ 45.9 kg ha^−1^) and was lowest at 45.9 kg ha^−1^ P, and the difference between the P_3_ and P1 treatments was significant (Table 1). This indicated that the addition of P adversely affected the PFPP. Similar results were found in a study by Fu et al.^13^, where the PFPP was reduced by P application measures. The recovery efficiency of fertilizers applied to the soil can be reflected in the fertilizer utilization efficiency^44,45^. The results of this study indicated that with the increase in P application, the PUE tended to increase and then decrease, and the PUE of *Leymus chinensis* in the P_1_ treatment was higher than that in the P_3_ treatment. This illustrated that the PUE can increase with an increase in P applied within a certain range, but beyond that range, it instead tends to decrease the PUE, even more so than the treatment with low P application. The PUE was even lower than that in the low-P treatment (P_1_), and the economic and environmental benefits should be considered during future fertilization processes. This is consistent with the findings of Xing et al.^46^ that excessive application of P resulted in lower yields and smaller economic benefits. Thus, reasonable application of fertilizer is fundamental for ensuring appropriate fertilizer utilization, and an increased application of P fertilizer within a certain range can promote nutrient uptake and accumulation in crops and thus improve the PUE^47,48^.

## Conclusions

Our results demonstrated that excessive phosphorus application is not beneficial for the growth of *Leymus chinensis* but will cause resources wastage and a series of negative impacts on the environment. Although the promotion effect was observed at the phosphorus application rate of 30.6 kg ha^−1^ and 45.9 kg ha^−1^, the promotion effect was not obvious at the phosphorus application rate of 45.9 kg ha^−1^, and the PUE was only 7.46%. Considering the economic cost, it is proposed to recommend the phosphorus application rate of 30.6 kg ha^−1^ for fertilization. However, this study was carried out in pots, the conditions of which differ from actual production conditions. In future studies, a large number of field trials should be carried out for further verification, and attention should also be given to combinations that optimize resource utilization and are environmentally friendly. Furthermore, in the application range of 0–30.6 kg ha^−1^, the PUE increased with the added amount of P fertilizer. However, the threshold value of the added amount of P fertilizer when the maximum PUE was reached needs to be further study.

## Materials and methods

### Study site and soil sampling

The experiment was conducted in the College of Resources and Environment of Xinjiang Agricultural University from May to October 2020. The tested soil comes from the experimental station of Xinjiang Agricultural University, Sanping Farm (43°56′ N, 87°35′ E, altitude 580 m), which is located at the northern foot of the Tianshan Mountains and the southern edge of the Zhunger Basin. The region belongs to the continental semi-arid climate, the annual climate of aridity and little rain, abundant sunshine and light resources, large temperature difference between day and night, long and cold winters, hot and dry in summers. Summer temperatures are highest in July and August when the average temperature reaches 23.0 °C. The annual average temperature is 8.7 °C, and the average annual precipitation is 199.6 mm, while the average annual evaporation is 2647 mm (http://xihe-energy.com) Physicochemical properties of experimental soil before the start of the experiment are shown in Table 2.

**Table 2:** Physicochemical properties of the experimental soil before the start of the experiment. AP, available P; OM: organic matter; AN, available nitrogen; AK, available K; TN, total nitrogen. Values in the table are mean ± standard error of the mean with the number of observation (n = 3).

Soil type	pH	AP (mg kg^−1^)	OM (g kg^−1^)	AN (mg kg^−1^)	AK (mg kg^−1^)	TN (g kg^−1^)
Calcareous soil	8.18 ± 0.21	13.33 ± 0.23	14.60 ± 0.03	45.14 ± 0.10	187.80 ± 0.21	0.62 ± 0.02

### Design of the pot experiments

In this experiment, *Leymus chinensis* (Zhongke No.1) was used as the test material, which was collected from the Hutubi experiment site of Xinjiang Jinfangyuan Grassland Ecotourism Development Co., LTD in 2019. The seeds were sieved before sowing to select fuller seeds, and seeds of poor quality were rejected to minimize the impact of poor seed quality on the test results. The soil was pre-treated, air-dried, de-mixed, and sieved before the pot culturing experiments, and the air-dried soil was weighed into pots (20 cm in diameter, 15 cm height) at 4.2 kg per pot. Weighed fertilizer was mixed thoroughly with the soil and added to the pots, and the seeds were sown after 24 h. The same number of seeds was sown into each pot. The seeds were sown at a depth of 2 cm, and the number of plants in each pot was maintained at 30 by thinning seedlings after they had stabilized. During the experiment, the trial factors were strictly controlled to eliminate errors as much as possible. Soil moisture was maintained according to weather changes and to be consistent between treatments.

The experiments were designed with a gradient of four P applications, treatment 1: P_0_, no P treatment; treatment 2: P_1_, 15.3 kg P ha^−1^ year^−1^; treatment 3: P_2_, 30.6 kg P ha^−1^ year^−1^; and treatment 4: P_3_, 45.9 kg P ha^−1^ year^−1^, with 6 replicates for each treatment. N fertilizer was used as the base fertilizer, and the same amount of N fertilizer was applied in each treatment to ensure that the basic plant nutrient requirements for N were met. The N fertilizer was applied at 150 kg N ha^−1^ year^−1^, and potassium fertilizer was not added during this experiment because of the high content of available potassium in the soil used in the experiment. The N fertilizers used in the experiment were urea (46% N) and ammonium dihydrogen P (26.64% P, 12% N), provided by Yunnan.

### Sample collection

In this experiment, soil samples and plant samples were collected during the jointing period (June 19, 2020) and during the harvest period (September 29, 2020) for *Leymus chinensis.* Sampling was carried out via a completely destructive sampling method: the soil was emptied from each pot and mixed, and then an appropriate amount of soil was collected using the quartation and passed through a 60-mesh sieve after natural air-drying. The soil chemical indices, such as available P and available N, were then measured. After the soil samples were separated from the roots of the plants, the plant was separated from the root and above ground, rained with deionized water and put into a Kraft paper bags, and oven-dried at 70 ℃ for 24 h. The dry weight of each part was measured and recorded, and the samples were crushed with a micro-crusher (FZ102) and used for plant nutrient measurements. Three biological replicates were set for each treatment at jointing period and harvesting period.

### Measurements

The measurement methods were based on "Soil Agrochemical Analysis"^49^.

(1) Measurements of the soil physical and chemical properties were conducted as follows. The pH of the air-dried soil was measured potentiometrically at a ratio (v/v) of 1:2.5 in distilled water, and then pH metre (FE22-Standard, Mettler, Shanghai) was used for pH determination. Soil organic matter (OM) was measured by an external heating method using potassium dichromate. Available P (AP) was determined by the sodium bicarbonate-molybdenum-antimony resistance calorimetric method. Soil available K (AK) was determined by ammonium acetate extraction-flame photometry. Available N (AN) was determined by the alkaline hydrolysis diffusion method. Soil total P (TP) was determined by the molybdenum-antimony resistance calorimetric method.

(2) Plant nutrients were measured as follows. Plant tissue was digested by the H_2_SO_4_–H_2_O_2_ method; then, plant total phosphorus (PTP) was determined by the vanadium molybdate yellow colorimetric method, and plant total nitrogen (PTN) was determined by the Kjeldahl method^50,51^.

### Calculation method

The indicators for P accumulation, P fertilizer partial productivity (PFPP) and PUE for each tissue of *Leymus chinensis* under the different P application conditions were calculated according to the following Eqs. ^46,52^:

1 $$PA = Dw \times Pc$$

2 $$NA = Dw \times Nc$$

3 $$PFPP = \frac{ Dw }{{Pa }}$$

4 $$PUE = \frac{{PA - P_{0} }}{Pa} \times 100\%$$

where PA and NA are the P accumulation and N accumulation (mg pot^−1^), respectively, Dw is the dry weight of the aboveground and belowground biomass, Pc and Nc are the percentages of P and N contents of the treatments, respectively, PFPP is the phosphorus fertilizer partial productivity; P_0_ is the P accumulation of control; Pa is the P application.

### Data analyses

The data of Xinjiang land-use type were obtained from GlobeLand30 2020 (http://www.globallandcover.com/). Meteorological data were obtained from the the Xihe Energy Big Data Platform (http://xihe-energy.com). The differences in soil available P, available N, biomass, and nutrient accumulation in the different organs of *Leymus chinensis* were tested using one-way analysis of variance (ANOVA). All ANOVAs were tested for significance at *p* < 0.05. The test data were compiled using Microsoft Excel 2007 and analysed for significant differences and correlation using SPSS 19.0 (IBM, Chicago, IL) statistical software, and graphical analyses were performed using Origin 2021 (Systat Software, Sanjose, CA). Principal component analysis (PCA) was conducted on biomass, nutrient accumulation, and soil available P and N during the different periods of *Leymus chinensis* growth. The data in the graphs are expressed as the mean (n = 3) ± standard error.

### Permission statement

This is a permission from College of Resources and Environment of Xinjiang Agricultural University to collect samples of *Leymus chinensis* (Zhongke No.1) from the Hutubi experiment site of Xinjiang Jinfangyuan Grassland Ecological Technology Development Co., LTD, were complied with relevant in-stitutional, nation, and international guidelines and legislation and transferring them safely to physicochemical analysis to Xinjiang Agricultural University, College of Resources and Environment. The *Leymus chinensis* (Zhongke No.1) used in the study was identified by National Grass Variety Council. The voucher specimen of the *Leymus chinensis* (Zhongke No.1) is not deposited in the public herbarium.

## Data Availability

The datasets generated and/or analyzed during the current study are available in the GlobeLand30 2020 (http://www.globallandcover.com/), and the Meteorological data were obtained from the Xihe Energy Big Data Platform (http://xihe-energy.com).
